# Unbalanced calcium channel activity underlies selective vulnerability of nigrostriatal dopaminergic terminals in Parkinsonian mice

**DOI:** 10.1038/s41598-019-41091-7

**Published:** 2019-03-19

**Authors:** Carmelo Sgobio, Lixin Sun, Jinhui Ding, Jochen Herms, David M. Lovinger, Huaibin Cai

**Affiliations:** 1 Transgenic Section, Laboratory of Neurogenetics, National Institute on Aging, National Institutes of Health, Bethesda, MD 20892 USA; 2 Translational research division, German Center for Neurodegenerative Diseases (DZNE), Munich, 81377 Germany; 3 Computational Biology Group, Laboratory of Neurogenetics, National Institute on Aging, National Institutes of Health, Bethesda, MD 20892 USA; 4 Center for Neuropathology and Prion Research, Ludwig-Maximilians-Universität München, Munich, 81377 Germany; 5 Laboratory for Integrative Neuroscience, Section on Synaptic Pharmacology, National Institute on Alcohol Abuse and Alcoholism, National Institutes of Health, Rockville, MD 20852 USA

## Abstract

Dopamine (DA) release in striatum is functionally segregated across a dorsolateral/ventromedial axis. Interestingly, nigrostriatal DA signaling disruption in Parkinson’s disease (PD) preferentially affects the dorsolateral striatum. The relationship between afferent presynaptic calcium transients (PreCaTs) in DA terminals and DA release in dorsolateral (*Caudato-Putamen*, DLS) and ventromedial (*Nucleus Accumbens* Shell, VS) striatal subregions was examined by e*x vivo* real-time dual-recording in conditional transgenic mice expressing the calcium indicator protein GCaMP3. In DLS, minimal increases in cytosolic calcium trigger steep DA release while PreCaTs and DA release in VS both were proportional to the number of pulses in burst stimulation. Co-expressing α-synuclein with the Parkinson’s disease (PD)-associated A53T mutation and GCaMP3 in midbrain DA neurons revealed augmented cytosolic steady state and activity-dependent intra-terminal calcium levels preferentially in DLS, as well as hyperactivation and enhanced expression of N-type calcium channels. Thus, unbalanced calcium channel activity is a presynaptic mechanism to consider in the multifaceted pathogenic pathways of progressive neurodegeneration.

## Introduction

The striatum has the richest dopamine (DA) innervation in the central nervous system (CNS), supplied by axon terminals of midbrain dopaminergic neurons (mDANs). The mDANs are unique in their extensive homeostatic regulation and axonal arborization^1^. Those mDANs that innervate the dorsolateral (DLS) and the ventromedial *Nucleus Accumbens* (NAc) shell (VS) striatum come from two distinct areas in the midbrain, the *Substantia Nigra pars compacta* (SNc) and the ventral tegmental area (VTA), respectively^2^. Dopamine regulates postsynaptic neuronal output, and there are functional differences in DA neuromodulation in different striatal subregions^3^. The nigrostriatal pathway targets DLS and is critical for movement and habit formation, while mesolimbic innervation in VS mediates reward and goal-directed responses^4^. Disrupted DA signaling in the striatum underlies basal ganglia circuit dysfunction and consequent movement disorders, including Parkinson’s disease (PD)^5^. As in many other neurodegenerative diseases, PD shows a selective neuronal vulnerability that involves a specific pattern of progressive degeneration. Interestingly, mDANs in the SNc are more susceptible to neurodegeneration when compared with the neurons in VTA^6^.

Because of the small dimensions and the massive distribution of the DA axonal ramifications in striatum, direct observation of terminals is presently challenging and mDAN somas are preferred as a more approachable target of such investigation. However, physiological analysis of axonal activity is crucial to understand the selective vulnerability of SNc mDANs and in general the progression of the neurodegenerative process. In fact, accumulating evidence shows that synaptic alterations at the terminal level precede the occurrence of neurodegeneration in genetic models of PD that overexpress α-synuclein, as well as in human cases^7–9^. So far, studies focused on the ability of mDAN terminals in DLS and VS DA axons to cope with PD-related insults are limited. It is important to understand intracellular calcium changes in presynaptic terminals as they provide crucial information about excitation-secretion coupling at a point upstream of neurotransmitter release^10^. To this end, we optimized a method for simultaneous DA measurement with Fast Scan Cyclic Voltammetry (FSCV) and presynaptic calcium transients (PreCaTs) using photometry in axons and terminals of mDANs^11^. The aim of this study is to understand the relationship between calcium activity in dopaminergic terminals and DA release in the DLS and VS, in normal and pathological conditions. For control mice, the genetically encoded calcium indicator GCaMP3 was selectively expressed in the mDANs under the transcriptional control of PITX3, using a binary tetracycline-dependent inducible gene expression system^11^. We then co-expressed the PD-related α-synuclein A53T mutation in the same mDANs together with GCAMP3. The resultant Pitx3-IRES2-tTA/tetO-GCAMP3/tetO-h-α-synA53T (A53T mutants) mice developed the same PD-like phenotype as described before^7^. In *ex vivo* slice dual recordings, DA release in DLS did not correlate with the calcium level reached after axonal activation, and even the lowest stimulation levels maximized DA release in this region. On the other hand, VS DA release was more tightly associated with PreCaTs amplitudes. In the A53T mutants there is a strong loss of DA release from terminals specifically in DLS. However, we observed for the first time the presence of high basal calcium levels in the axons in this PD model, as well as significantly higher PreCaTs in response to a number of different stimulation protocols. Enhanced inhibition by inhibitors of N-type calcium channel inhibitors and reduced effects of P/Q-type calcium channel blockade on DLS PreCaTs suggests that enhanced N-type channel function may contribute in part to the augmented calcium availability in these terminals, indicative of a mechanism that might promote axonal degeneration.

## Results

### PreCaTs and DA release are differentially interrelated in ventral and dorsal striatal subregions

**Figure 1:**
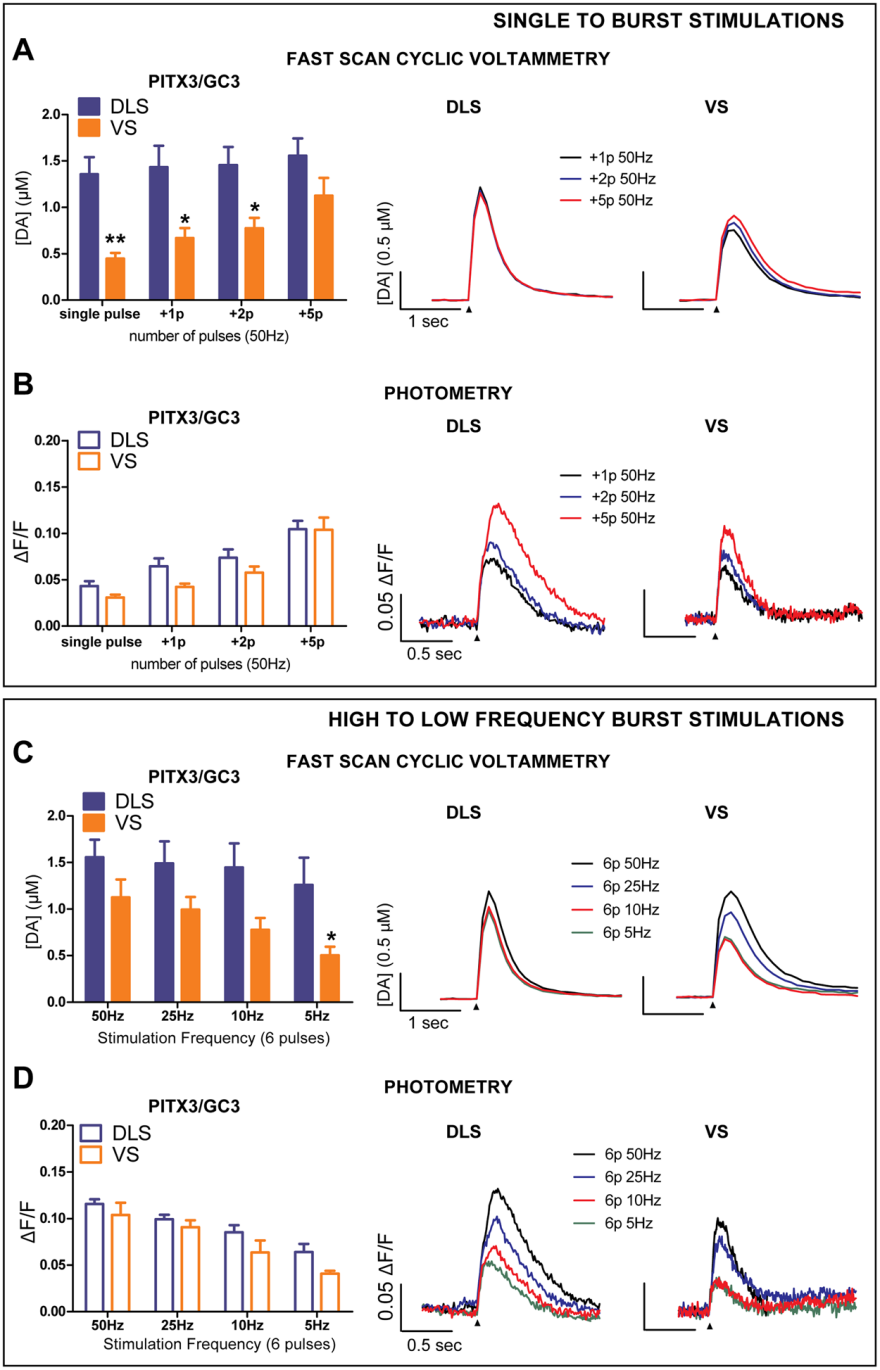
Differences between DLS and VS DA release were not accompanied by corresponding changes in PreCaTs in PITX3/GC3 mice. (**A**) DA release in DLS was not affected by the number of pulses of electrical stimulation, while in VS release was pulse number dependent. Two-way ANOVA Area X Pulse Number INTERACTION F_(3,24)_ = 5.794; p < 0.01. Bonferroni Post Hoc test: *p < 0.05, **p < 0.01. (**B**) PreCaTs in both regions increased proportionally with the number of the pulses delivered. Two-way ANOVA Pulse Number MAIN EFFECT F_(3,24)_ = 62.71, p < 0.001. (**C**) Changing the frequency of 6-pulse burst stimulation strongly increased DA release in VS, but not in DLS. Two-way ANOVA Area X Frequency INTERACTION F_(3,24)_ = 3.288, p < 0.05. Bonferroni Post Hoc test: *p < 0.05. (**D**) PreCaTs in both DLS and VS increased in amplitude with increasing intra-burst frequency. Two-way ANOVA Frequency MAIN EFFECT F_(3,24)_ = 34.02, p < 0.001. Representative traces of PreCaTs are shown at right in each of the figure panels.

The recordings of DA release acquired in our conditional GCaMP3 mice confirmed the neurotransmission dynamics that differentiate DA fibers in DLS from VS^12^. In fact, after increasing the number of pulses at the same stimulation intensity and frequency (50 Hz), DA transients in DLS did not significantly increase in amplitude. However, DA release from VS fibers was proportional to the number of pulses within a 50 Hz burst stimulation (Fig. 1A). This difference was not observed in concurrently measured PreCaTs, where the amplitudes increased proportionally with the number of the pulses in both regions (Fig. 1B). A similar discrepancy between DA release and calcium transients was also observed when the frequency of stimulation within the same burst (6 pulses) was changed from 50 Hz to 5 Hz. The inter-regional divergence in DA release was observed for DA (Fig. 1C), but PreCaTs were still comparable (Fig. 1D). These results showed how minimal influx of calcium within the DLS triggers strong DA release, while in VS the DA release was moderate and proportional to the calcium transient within the stimulation paradigms used.

### D2R activation strongly affects DA release, but the effects differ across striatal regions for concurrently-measured PreCaTs

**Figure 2:**
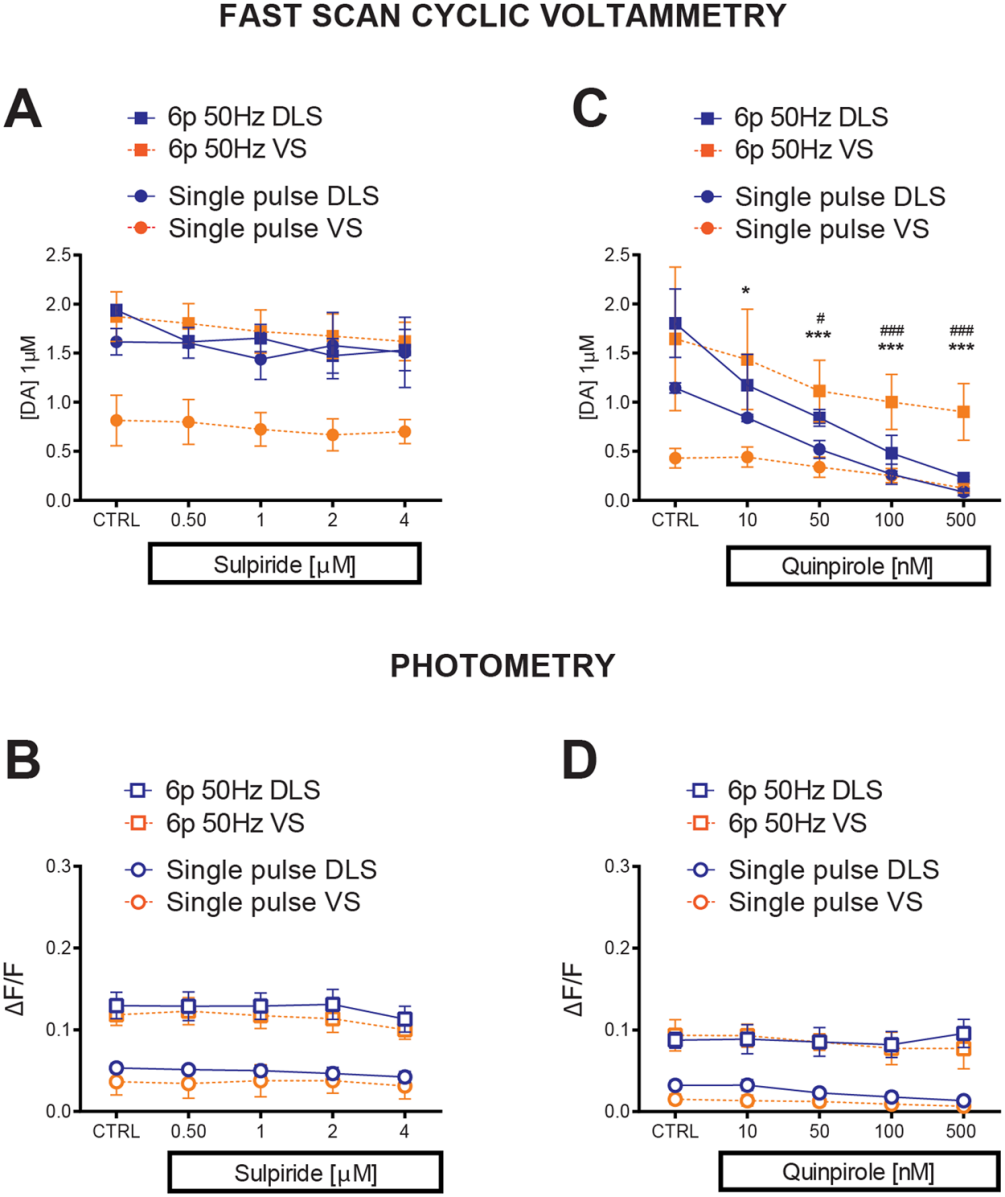
Pharmacological manipulations of D2R affect DA release, but effects differ across striatal regions for concurrently-measured PreCaTs. (**A**) For DA release and (**B**) PreCaTs, the D2R antagonist sulpiride had no effect in either striatal region, with either single or 6-pulse burst stimulation. (**C**) The D2R agonist quinpirole decreased DA release in a dose-response manner, with higher efficacy in DLS compared to VS. This effect was not observed for their relative PreCaTs (“Single pulse”: Two-way ANOVA Area X Dose INTERACTION F_(4,16)_ = 11.65 p < 0.001. “Burst 6p50Hz”: Two-way ANOVA Dose MAIN FACTOR F_(4,16)_ = 11.74 p < 0.001). (**D**) Where a dose-dependent decrease was detected for single pulse stimulation (“Single pulse”: Two-way ANOVA Dose MAIN FACTOR F_(4,16)_ = 7.202 p < 0.01. “Burst 6p50Hz”: Two-way ANOVA not significant).

DA release and availability in the extracellular space are regulated by feedback mechanisms on the presynaptic DA terminal. The G_i/o_–coupled inhibitory Dopamine D2 autoreceptors (D2Rs) suppress DA release^13^. For pharmacological manipulations of D2R, increasing antagonist or agonist concentrations were perfused consecutively in the bath for 20 min each and changes in amplitudes were recorded using single pulse or burst (6 pulses at 50 Hz) stimulation. The D2R antagonist sulpiride had no effect in either striatal region on either DA release or PreCaTs induced by single stimulus pulses or bursts (Fig. 2A,B). The lack of effect on stimulus-evoked DA release is probably due to the absence of tonic DA levels in the slice preparation, together with the low level of DA release evoked by the stimulating protocol we applied. However, a robust effect on dopamine release was found after the application of the D2R agonist quinpirole, with higher efficacy in DLS compared to VS (Fig. 2C). This effect was not as strong for the PreCaTs in these regions, as only a mild dose-dependent decrease was detected for single pulse stimulation, but not burst stimulation (Fig. 2D). These data are consistent with the idea that presynaptic calcium levels may be related to D2R effects on DA release during low frequency activation, but are not directly involved in the auto-regulatory feedback mechanisms within presynaptic DA terminals at higher activation frequencies.

### Opposite effects of paired-pulse stimulation differentiate VS from DLS PreCaT dynamics

**Figure 3:**
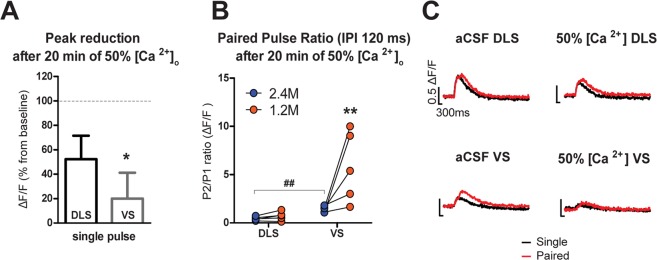
Paired pulse stimulation resulted in PreCaTs inhibition in DLS, and facilitation VS with high sensitivity to [Ca^2+^]o changes in VS. (**A**) Reducing aCSF calcium concentration from 2.4 mM to 1.2 mM strongly decreased PreCaTs in VS, with a smaller effect in DLS, in the single pulse stimulation paradigm (Student’s t test: t_(8)_ = 2.529, p < 0.05). (**B**) The same calcium concentration reduction facilitated DA release during paired pulse stimulation in VS, but not in DLS (Two-way ANOVA Area X Concentration Interaction F_(1,8)_ = 5.895, p < 0.05 Bonferroni Post Hoc test **^,##^p < 0.01). (**C**) Representative PreCaTs traces of reduced calcium paired pulses experiment.

To determine if there is a subregional difference in striatal PreCaTs dependence on extracellular calcium concentration ([Ca^2+^]_o_), we compared the sensitivity of PreCaTs to a 50% [Ca^2+^]_o_ reduction in our aCSF (from 2.4 mM to 1.2 mM) using single pulse and paired-pulses protocols (Fig. 3A–C). We observed that DLS terminals showed smaller decreases in single-pulse evoked PreCaTs following the decrease in [Ca^2+^]_o_ compared to VS fibers, indicating weaker dependence on extracellular calcium in DLS (Fig. 3A). The paired-pulse stimulation protocols were employed to test the ability of presynaptic terminals to regulate transient elevation of intracellular calcium in response to two action potentials occurring in close temporal contiguity. Using an inter-pulse interval (IPI) of 120 ms in DLS, we observed a substantial paired-pulse inhibition, in contrast to the paired pulse facilitation observed in VS (Fig. 3B,C). Lowering aCSF [Ca^2+^] increased the PPR significantly only in VS (Fig. 3B,C). These results indicate that DA terminals in DLS and VS differently handle intracellular calcium following terminal depolarization. Furthermore, the ability to DA neurotransmission is subregion specific.

### A53T α-synuclein mutation mainly affects DLS DA release, resulting in an increase in calcium influx dependence

**Figure 4:**
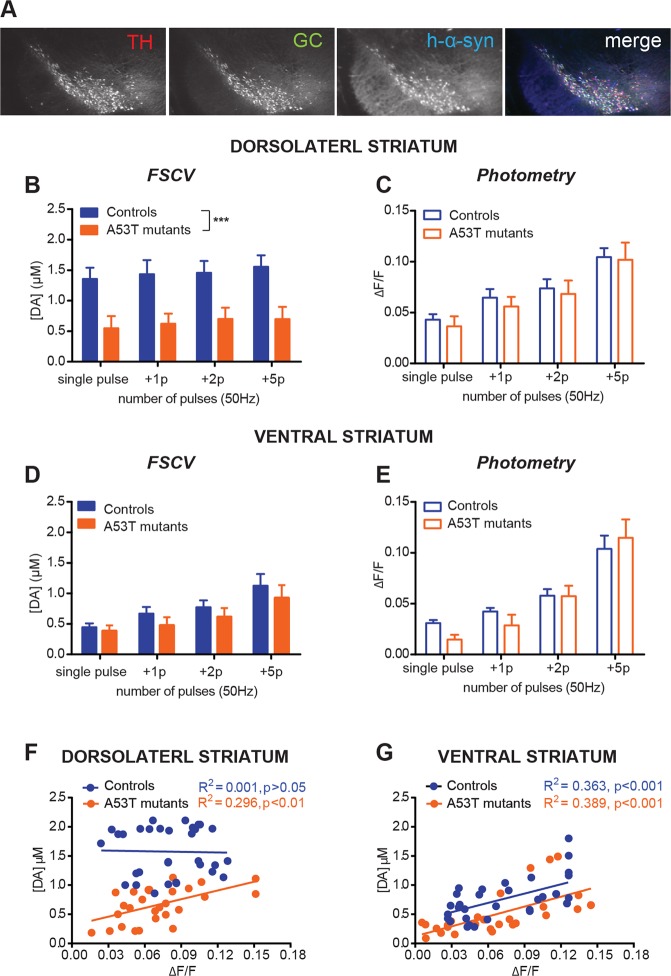
Mice carrying the A53T PD-related α-synuclein mutation show DLS-specific reduction of DA release but no difference in PreCaTs. (**A**) Representative images show the distribution of tyrosine hydroxylase (TH), human mutated A53T α-synuclein protein (h-α-syn) and GCaMP3 (GC) proteins in midbrain coronal sections of 3 month-old PITX3/GC/A53T (A53T mutants) mice (Two-way ANOVA Genotype MAIN FACTOR F_(1,7)_ = 8.541). (**B**) DA release in DLS in both genotypes did not increase with increasing number of pulses in a burst. Strong impairment of release was observed in DLS of A53T mutant mice at all burst durations (ANOVA Genotype Number MAIN FACTOR F_(1,28)_ = 33.24, p < 0.001). (**C**) Corresponding photometry recordings revealed that PreCaTs increased in proportion with the number of pulses in both genotypes in DLS (ANOVA Pulse Number MAIN FACTOR F_(3,28)_ = 13.48, p < 0.001). (**D**) In VS, DA release increased with increasing burst duration, but did not differ between controls and A53T mutant slices (ANOVA Pulse Number MAIN FACTOR F_(3,28)_ = 7.20, p < 0.001), (**E**) as well as for corresponding PreCaTs (ANOVA Pulse Number MAIN FACTOR F_(3,28)_ = 31.41, p < 0.001). Scatterplot comparing PreCaTs and [DA] release in DLS revealed a significant correlation in A53T mutants but not in Control mice (**F**) (Pearson’s r = −0.02522, R^2^ = 0.0006 p > 0.05; A53T mutants: Pearson’s r = 0.5437, R^2^ = 0.2956 p < 0.01). Same scatterplot for VS showed significant correlation in both A53T mutants and Control mice (**G**) (Controls: Pearson’s r = 0.6024, R^2^ = 0.3629 p < 0.001; A53T mutant: Pearson’s r = 0.6239, R^2^ = 0.3893 p < 0.001).

To enhance our understanding of potential PD-related changes in DA axon terminals, we co-expressed the PD-related α-synuclein A53T mutation in the same mDANs with GCAMP3 by crossbreeding three different lines of transgenic mice (Fig. 4A). Simultaneous DA release/PreCaTs recordings from A53T mutant mice revealed the same stimulus-dependence for increased DA as observed in control littermates. However, a dramatic impairment in DA release in DLS was observed (Fig. 4B), as previously reported^7^, concurrent with no alteration of PreCaTs amplitudes in A53T mutants compared with control littermates (Fig. 4C). This pattern of decreased DA release with no change in PreCaTs was observed in response to both single-pulse and burst stimulation. The same DA release and calcium dynamics discrepancy was also observed when the stimulation frequency within a 6 pulse burst was decreased from 50 Hz to 5 Hz (Supp. Fig. S1). Unlike in the DLS, in the VS no decrease in DA transient amplitude was observed (Fig. 4D). However, the pattern of PreCaTs was similar to that observed in DLS, especially in response to the longest burst stimuli (Fig. 4E). Scatter plots of the same data revealed a significant correlation between PreCaTs and DA release in DLS of A53T mutant mice but not in the controls (Fig. 4F). However, in VS the correlations were similar in both genotypes (Fig. 4G). These experiments revealed a strong impact of the A53T on DA release only in DLS, but no accompanying change in PreCaTs in either region. In this case, A53T mutant-containing DA terminals showed evidence of a relationship between DA release and PreCaT amplitude, differing from DLS features observed in the controls.

### A53T PD-related α-synuclein mutation led to augmented calcium levels despite reduced axonal arborization

**Figure 5:**
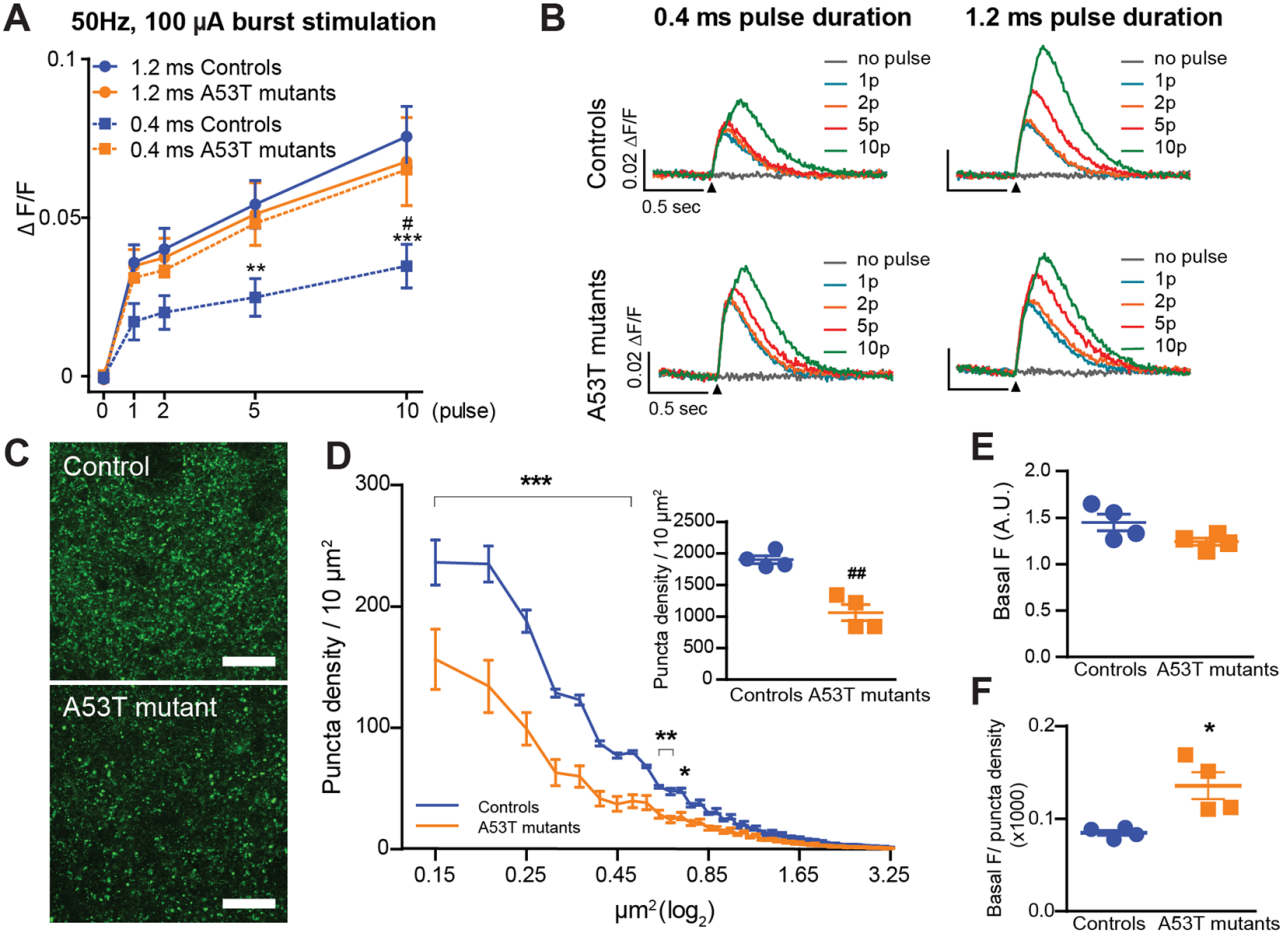
Expression of the A53T PD-related α-synuclein mutation led to augmented calcium levels in spite of reduced axonal arborization. (**A**) Single to burst stimulation I/O curve using different pulse durations and corresponding PreCaTs traces in PITX3/GC (Controls) and A53T mutant mice, and (**B**) representative traces. Increases in transient amplitude from single to multiple pulse stimulation were observed in both Controls and A53T mutants mice. However, only in control mice the amplitude proportionally increased with pulse duration. Indeed, 0.4 ms pulse-induced transient amplitudes were comparable to the 1.2 ms pulse responses recorded in mutant mice (Two-way ANOVA Genotype X Pulse Number INTERACTION: Controls: for 1.2 ms duration: F_(1,14)_ = 20.21, p < 0.001; for 0.4 ms duration, F_(1,14)_ = 5.71, p < 0.05. A53T mutants: 1.2 ms duration: F_(1,14)_ = 8.93, p < 0.01; 0.4 ms duration: F_(1,14)_ = 10.17, p < 0.01. Two-way ANOVA Stimulus Duration X Pulse Number INTERACTION: Controls: F_(4,24)_ = 11.06, p < 0.001. A53T Mutant: not significant. Bonferroni Post Hoc test within control group: **p < 0.01, ***p < 0.001. Between genotype (duration: 0.4 ms): ^#^p < 0.05). (**C**) GFP staining in DLS of 3-month-old Control (PITX/GC) and A53T mutant (PITX3/GC/A53T) mice. Scalebar = 10 µm. (**D**) Distributions of GFP-positive puncta density for different areas in tissue collected from the same mice used for photometry (with average densities in inset). A significant reduction of observed frequency of observing puncta smaller than 0.6 µm^2^ was detected in A53T mutants in the DLS (Two-way ANOVA Genotype X Puncta dimension INTERACTION: F_(78,468)_ = 15.4, p < 0.001; Bonferroni’s post hoc tests: *p < 0.05,**p < 0.01, ***p < 0.001. Student’s t test: t_(6)_ = 5.845, ^##^p < 0.01).

To sort out the origin of the discrepancy between intracellular calcium and DA release, we focused on the effect of different burst stimulation protocols on PreCaTs localized in DLS. For a better signal to noise ratio, we used fluorescence excitation exposures of 5-sec duration recorded every 30 sec, and then averaged traces every 5 sweeps, as reported previously^11^. Repeated burst stimulation (50 Hz, 100 µA) with different numbers of pulses resulted in a linear increase of the PreCaT amplitudes in both genotypes (Fig. 5A,B). In the control condition, changing the pulse durations of the stimulation (1.2 ms to 0.4 ms) led to a significant decrease in the magnitude of the PreCaTs (Fig. 5A,B). However, DA terminals in A53T mutant mice lost duration sensitivity, showing the same enhanced signal for both stimulation protocols (Fig. 5A,B). This result showing increased calcium changes in the presence of the α-synuclein mutation prompted us to extend our analysis to immunostaining of DA afferents and terminals in the same DLS slices used for burst-induced PreCaTs recording (Fig. 5C). Our data show a significant decrease of GFP-positive puncta (presumed to be dopaminergic varicosities) in striatum in A53T mutants, especially for puncta smaller than 0.6 µm^2^ (Fig. 5D), as well as a decrease in the percent area covered by labeled material (Controls: 9.88 ± 1.18 puncta, A53T mutants: 5.61 ± 0.28 puncta; t_(6)_ = 3.5, p < 0.05, data not shown). Reduced DA innervation in DLS was confirmed by analyzing TH-positive area coverage in non-GCaMP3 court of A53T mutants and littermates. In contrast in VS, the averaged area covered by the TH-positive puncta was comparable between A53T and WT, although there was a different in size composition in their puncta frequency distribution (Supp. Fig. S3). Altogether, these *ex vivo* data report for the first time the presence of high stimulus-induced calcium increases in the surviving axon terminals in a PD model, correlated with a lack of sensitivity to increasing action potential numbers and stronger responses to electrical stimulation.

### DLS-specific unbalanced activity between N-type and P/Q type calcium channels in A53T mutant mice

**Figure 6:**
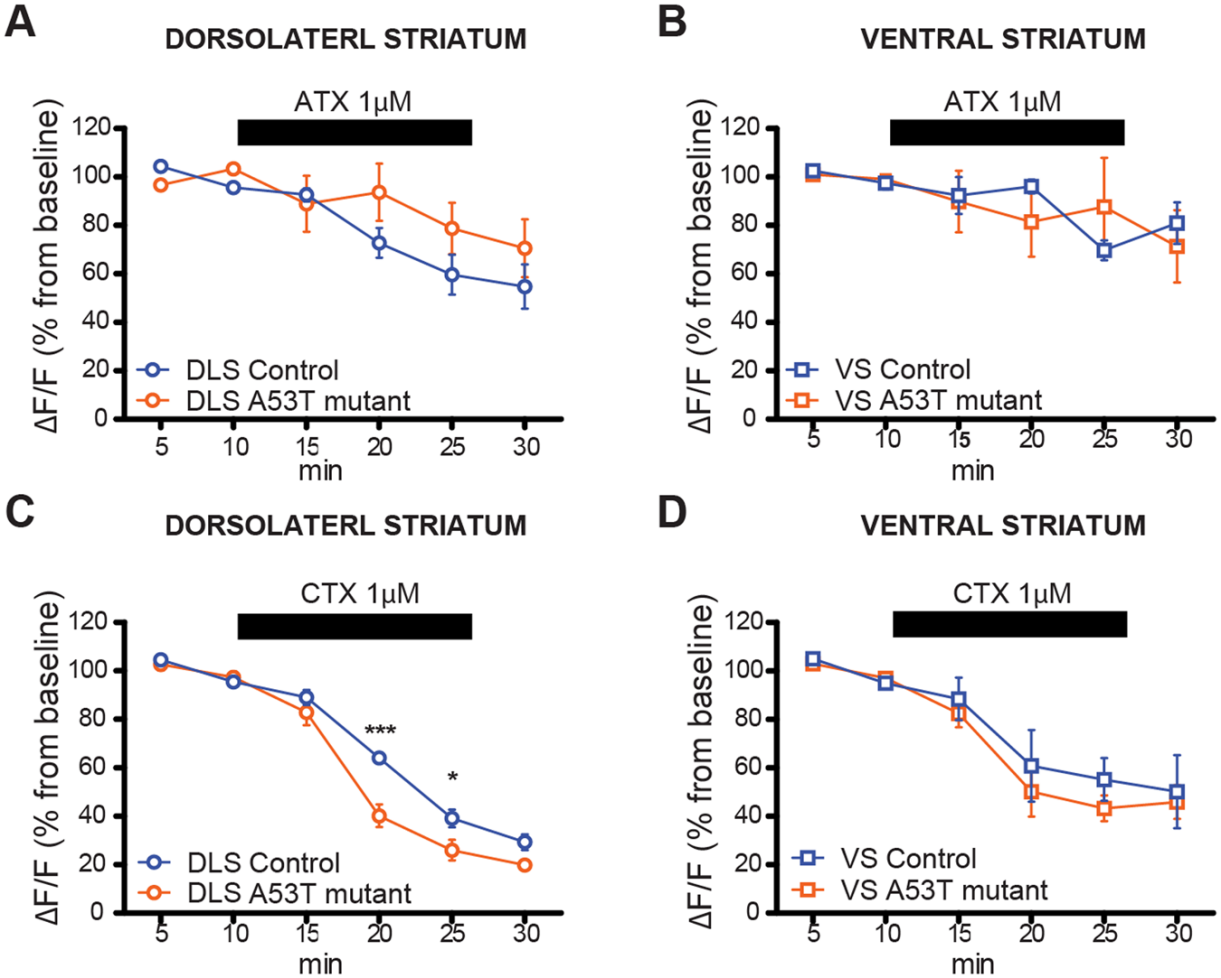
DLS-specific unbalanced activity between N-type and P/Q type calcium channel contribution to PreCaTs in A53T mice versus controls. The P/Q type calcium channel blocker ω-agatoxin IVA (ATX) decreases PreCaTs to a similar extent in both genotypes, but a slower onset of the effect was observed in A53T mutant DLS slices (**A**) (Two-way ANOVA Time X Genotype INTERACTION F_(5,40)_ = 2.511, p < 0.05) but not in VS slices (**B**) (Two-way ANOVA Time MAIN FACTOR F_(5,40)_ = 3.456, p < 0.05). In contrast, a faster onset and larger overall inhibition was observed during application of ω-conotoxin GVIA (CTX) a specific blocker for N-type calcium channels, in A53T mice relative to controls in DLS (**C**) (Two-way ANOVA Time X Genotype INTERACTION F_(5,40)_ = 4.546, p < 0.01. Bonferroni’s post hoc test, *p < 0.05. ***p < 0.001) but not in VS (**D**) (Two-way ANOVA Time MAIN FACTOR F_(5,35)_ = 38.04, p < 0.05).

To understand if the hyperactivation of A53T mutant DA terminals involves dysfunction of calcium influx through specific voltage-gated calcium channels (VGCCs), we examined effects of calcium channel blockers on PreCaTs recorded from A53T mutant mice and their control littermates, in both DLS and VS. We previously reported that blockers of N- and P/Q-type VGCCs were the only ones that inhibited PreCaTs in DLS during single pulse stimulation^11^. We now report that T-type VGCC blockade had no effect on PreCaTS in either DLS or VS (Supp. Fig. S2). Furthermore, we did not observe any effects of T-type or L-type VGCC blockers on PreCaTs in WT mouse slices using single pulse stimulation (Supp. Fig. S2). In our photometry experiments, the P/Q type VGCC blocker ω-agatoxin IVA (ATX, 1 µM) decreased PreCaT amplitudes to a similar extent in both genotypes. However, a slower onset of the effect of ATX in A53T mutant slices was observed in DLS (Fig. 6A) and not in VS (Fig. 6B). In contrast, a faster onset and larger overall inhibition was observed after application of ω-conotoxin GVIA (CTX, 1 µM), a specific blocker of N-type VGCCs, in A53T mice relative to controls, in DLS (Fig. 6C); but not in VS (Fig. 6D). In conclusion, these data revealed altered contribution of VGCCs to calcium influx in DLS presynaptic DA terminals of A53T mutants, where there is an enhanced role of N-type channels.

### The cytosolic calcium increase during paired-pulse stimulation is reduced in A53T mutants

**Figure 7:**
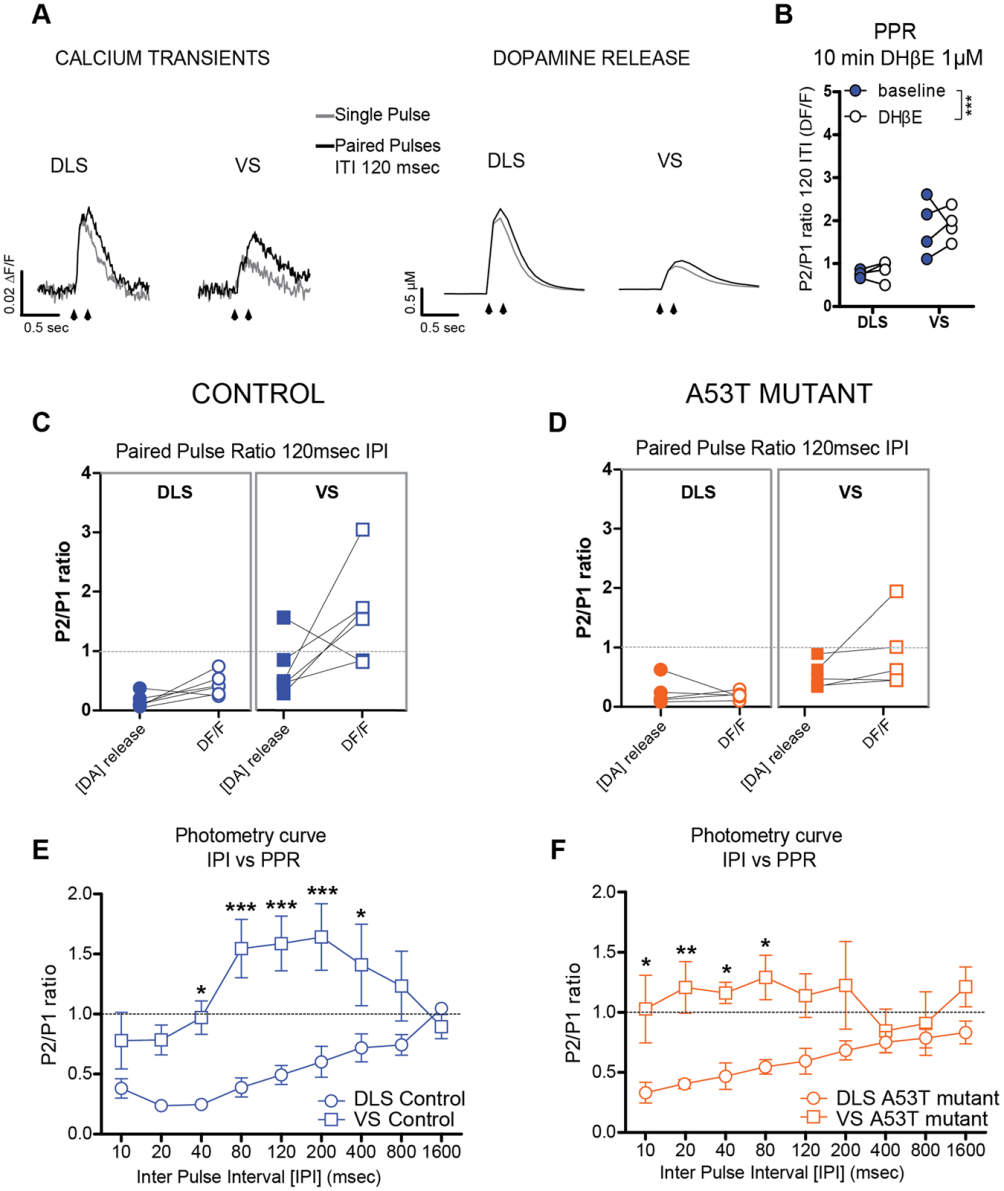
Paired Pulse Ratio (PPR) revealed differences in cytosolic calcium control between DLS and VS presynaptic DA terminals, and reduction of PPR in A53T mutants. (**A**) Representative traces of calcium transients (left) and DA release (right) with single and paired pulse stimulation (IPI 120 msec). (**B**) The regional difference in PPR of PreCaTs in control was maintained in the presence of an nAChR antagonist Two-way ANOVA Area MAIN FACTOR _F(1,28)_ = 33.24, p < 0.001). (**C**) Simultaneous photometry and voltammetry paired pulse recordings show depression in DLS for both DA release and PreCaTs, with a slight depression of DA release, but facilitation of PreCaTs in the VS. (**D**) A53T mutants showed profound paired-pulse depression in both DA release and PreCaTs in DLS, and a mild reduction in PreCaTs PPR in VS. Detailed analyses of the time course of PPR at different inter-pulse intervals (IPI) with photometry only (Two-way ANOVA Area x Genotype comparisons. For DA release: Area MAIN FACTOR F_(1,18)_ = 10.67, p < 0.01; For Calcium transients: Area MAIN FACTOR F_(1,18)_ = 17.02, p < 0.001; Genotype Main Factor F_(1,18)_ = 4.447, p < 0.05). Paired pulse facilitation was observed in control VS (**E**) for IPIs shorter than 1 sec, while in DLS depression was observed at all IPIs shorter than 1.6 sec. A similar trend was observed in A53T mouse slices (**F**), but with a moderate reduction of PPR in VS (Two-way ANOVA Genotype x IPI INTERACTION in VS: _F(8,72)_ = 2.368, p < 0.05. No significant post hoc).

As described before^14^, the paired-pulse stimulation protocol provides information about regulation of calcium influx in response to repeated stimulation of axon terminals (Fig. 7A). It is important to note that the regional difference in PPR of PreCaTs in control was maintained in the presence of an nAChR antagonist (Fig. 7B). This indicates that any effects due to activation of cholinergic stimulation by electrical stimulation were not involved in the PreCaTs dynamics we observed. Simultaneous photometry and voltammetry paired pulse recordings indicate depression in DLS for both DA release and PreCaTs, while a slight depression of DA release, but facilitation of PreCaTs was observed in the VS. A53T mutants showed profound depression of DA release and PreCaTs in DLS, and also a mild reduction in PreCaTs PPR in VS (Fig. 7C,D). In a detailed photometry analyses of PPR, a range of different IPI between 10 and 1600 ms showed a paired pulse facilitation in control VS for IPIs shorter than 1 sec (Fig. 7E). Meanwhile, in DLS depression was observed at all IPIs shorter than 1.6 sec. A similar trend was observed in A53T mouse slices, but with a moderate reduction of PPR in VS (Fig. 7F). These results confirmed an A53T-induced deficit in intracellular calcium management in DA terminals, with a significantly higher magnitude for terminals located in DLS.

### SNc mDANs show early upregulation of N- and T-type VGCC gene expression

**Figure 8:**
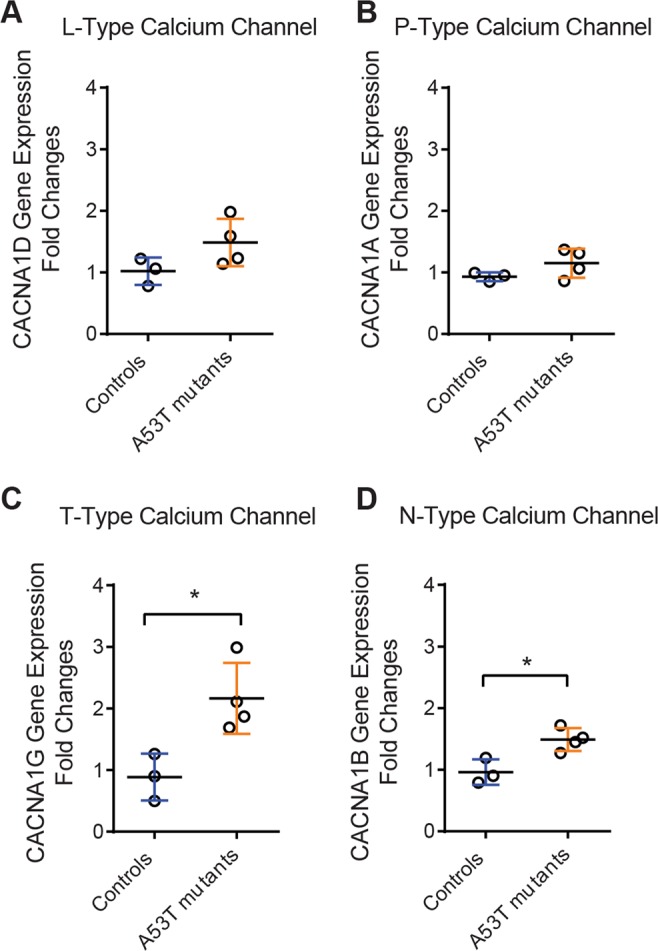
LCM/qRT-PCR mRNA measurement in 1-month-old mDA neurons revealed early augmented calcium channel transcription pattern. (**A**) L-type calcium VGCC subunit alpha1 D (CACNA1D) gene showed no significant change in A53T mutant mice. (**B**) For calcium VGCC subunit alpha1 A (CACNA1A), which encodes P-type channels, expression in mutants was similar to that observed in control mice. (**C**) Analysis of calcium VGCC subunit alpha1 G (CACNA1G), which encodes T-type channels, showed a robust 2-fold increase in mRNA transcription in the A53T mutant overexpressing mice (Student’s t test: t_(5)_ = 3.567, p < 0.05). (**C**) N-type VGCCs, encoded by calcium voltage-gated channel subunit alpha1 B (CACNA1B) showed a significant upregulation in A53T mutants (Student’s t test: t_(5)_ = 3.301, p < 0.05).

To determine if VGCC subunit expression was altered in A53T mutant-expressing SNc mDANs relative to controls, we performed laser capture microdissection (LCM) and qRT-PCR analyses of mRNA for different calcium channels using tissue collected from 1-month-old mice (a time point prior to strong pathology). For alpha1D (CACNA1D), the gene that encodes the L-type VGCC, we observed no change in A53T mutant SNc relative to WT mice (Fig. 8A). The alpha1A (CACNA1A) subunit, which encodes P-type VGCCS, showed comparable levels in both genotypes (Fig. 8B). Analysis of the alpha1B subunit (CACNA1B) of the N-type VGCC showed a significant upregulation in A53T mutants (Fig. 8C), with a similar upregulation of the T-type VGCC, encoded by subunit alpha1G (CACNA1G) (Fig. 8D). These results revealed an early upregulation of key calcium channel expression in SNc DANs before substantial neuronal loss due to A53T mutant α-synuclein overexpression.

## Discussion

The findings described in the present work revealed region-specific dynamics of presynaptic calcium modulation in DA axon terminals under both physiological and PD-related pathological conditions. Aberrant striatal dopaminergic transmission contributes to various neurological disorders. A better understanding of why dopaminergic dysfunction is specific to the dorsolateral portion of the striatum is a fundamental step in the understanding of PD pathology and possibly in the development of future therapeutic strategies to treat this disorder. Molecular and electrophysiological discrimination of differences between SNc and VTA mDANs at the somatic level establish most of our understanding of the factors related such differential vulnerability^15^. Anatomically, these two sub-regions receive innervation from overlapping but distinct projecting areas^16^, and DA release modulation through D2R autoreceptors is enhanced in SNc compared with VTA mDA neurons^17^. The same specific DA release and uptake differences also appear to be present within efferent projections, as SNc and VTA innervate the striatum in a complementary way, following a dorsal to ventral gradient pattern highly conserved across different species^18^. To investigate possible physiological and pathophysiological differences in DA terminals within the two efferent striatal projections, we combined techniques for simultaneous fast scan cyclic voltammetry and photometry dual recording in slices of DLS and VS^11^, allowing us to examine the relationship between DA release and PreCaTs.

Interestingly, we observed that minimal increases in cytosolic calcium were sufficient to trigger steep DA release from DLS, while in VS the quantity of DA released was moderate and proportional to the calcium transient over the stimulation intensities used. Another confirmation that dorsal and ventral striatal DA terminals show differential relationships between intracellular calcium and DA release came from paired pulse experiments. The dissociation between paired-pulse inhibition in DLS versus facilitation in VS was also confirmed by the augmented sensitivity to reduced extracellular [Ca^2+^] for both DA release and PreCaTs, implying that DLS terminals are constantly exposed to higher cytosolic calcium levels than DA terminals in VS. The calcium-binding protein calbindin-D_28K_ (CB) has been proposed as a resilience factor in DA neurons and their axon terminals^19^. Efficiently buffering calcium might therefore reduce vulnerability to mitochondrial weakening, ultimately conferring resistance to PD-related damage^20^. The distinctive abundance of calcium buffer proteins in the ventral DA axons and the lower levels in dorsal striatum might explain the distinctive calcium dependency of DA release. However, the lack of calcium buffer expression by itself is not sufficient to confer resistance or sensitivity to degeneration in DLS, since the DA fibers within the Nucleus Accumbens shell do not express high buffering capacity^21^, but are relatively spared in PD and PD models. Our dual recordings in VS support this lower sensitivity to PD-related pathology, as we did not observe any significant impact of the overexpression of A53T mutation on DA release capability or the relationship to PreCaTs. Nonetheless, evidence supports the idea that calcium dysregulation is a major factor that leads to early synaptic failure in PD^22^. Because of the energetic demand of maintaining ion gradients for synaptic transmission, excitability is a pivotal characteristic of the physiology of mDANs and their vulnerability to damage^23^.

Mutations of the α-synuclein gene lead to early-onset autosomal-dominant familiar forms of PD. α-synuclein aggregation has been found in Lewy bodies in specific locations of the brain^24^, and are used as a PD marker. High levels of α-synuclein have been reported in PD patients^25^ and increasing evidence shows how elevated intracellular calcium promotes α-synuclein aggregation^26^, while compromising calcium homeostasis and mitochondrial integrity probably due to soluble oligomer toxicity^27^. Aiming to expand the investigation of PD-related pathology to include direct measurement of the function of DA axon terminals *in situ*, we co-expressed the PD-related α-synuclein A53T mutation in the same mDANs together with GCaMP3. The resultant conditional transgenic mice developed the same PD-like phenotype described before^7^. In particular, adult A53T mutant mice developed profound motor disabilities and robust mDAN neurodegeneration in SNc but moderate degeneration in VTA together with decreased striatal DA basal levels as well as DA release impairment in dorsal striatum. In this work, we extended the investigation of DA release to the ventral area of the striatum of this transgenic model of PD, and in spite of mild but significant loss of VTA mDANs and a marginal alteration in the size distribution of dopaminergic presynaptic boutons, we did not observe any severe impairments of DA neurotransmission. On the other hand, we observed a remarkable discrepancy between the dramatic reduction of DA release in dorsal striatum and the substantial hyperactivation of presynaptic calcium responses to electrical stimulation. It has been shown that the A53T mutation interferes with neurotransmitter release by slowing vesicle fusion, perhaps due to α-synuclein promotion of fusion pore dilation^28^ and inhibition of slow and rapid endocytosis^29^. Increased calcium in axons could be a compensation^30^ that works to restore proper DA release levels in striatum during PD pathogenesis. Specifically, reduced DA release might decrease the dopamine autoreceptor DRD2-mediated inhibitory activity, which in turn enhances VGCC-dependent calcium transients^31^. In addition, the involvement of mitochondrial ROS production mediated by increased cytosolic calcium levels could be one of the essential steps in the vicious cycle that promotes neurodegeneration^32^.

Using single pulse stimulation, we found that N-type calcium channels affect DA release in both areas of the striatum, but P/Q-type channel blockers produced larger decreases in DA release in DLS relative to VS. We then found that A53T mutation overexpression led to DLS-specific unbalanced activity between N-type and P/Q type VGCCs. In fact, a faster onset and larger overall inhibition was observed after application of CTX, a specific blocker for N-type VGCCs, concomitant with a slower onset of the effect of ATX on P/Q VGCCs in A53T mutant slices. These results follow other evidence in the literature showing how exogenous α-synuclein selectively activates N-type channels extracellularly, reducing its raft partitioning and increasing DA release, both *in vitro* and *in vivo*^33^. Our PD-model expresses the A53T mutation in cytosol, indicating possible effects on the intracellular mechanisms in mDANs, possibly including components of N-type channels. It is possible that the potentiation of the N-type channel involves extracellular mechanisms, as mutant protein may spill out of damaged DA axons in our 3 month old mice. To determine if N-channel functional changes might involve mechanisms intrinsic to mDANs, we analyzed calcium channel transcription patterns in younger mice. Our LCM/qRT-PCR readout from 1-month-old mDANs revealed a change in calcium channel transcription. First, we observed a general trend for increased expression for all the main calcium channel genes. In particular, T-type channel mRNA levels from A53T mice were 2-fold higher compared to control mDA neurons. However, inhibiting T-type channel during single pulse stimulation did not reveal any significant role for this channel in PreCaTs in DLS. This was also observed for DA release, although 100hz burst stimulation unmasked a moderate decrease of DA release amplitude after blocking T-type receptors^34^. The study of the T-type channel role in presynaptic DA terminal has been shown to be challenging^35^ and more investigation may be required to understand if this channel contributes significantly to the regulation of striatal dopamine release under physiological conditions. More interestingly, N-type calcium-channel mRNA levels increased significantly in A53T mutant mice, supporting the hypothesis that the N-type channel might be already hyperactive at the terminals before any significant loss of dopaminergic neurons. Thus, the augmented N-type channel activity should be considered as a crucial pathological factor that probably originates from a homeostatic adaptation not only locally at the surviving DA terminals, but also involving early regulation at the nuclear level. To our knowledge, there are no direct interaction between N- and T-type calcium channels in dopaminergic level. Both of them have impact on pacemaker regularity but through different mechanisms^36^. However, it is indicative that in our A53T mutants a presymptomatic overexpression of these calcium channels precede the onset of the cell loss. Interestingly, N- and T-type calcium channel antagonist NP078585 attenuates or abolishes the acute effects of ethanol and abnormal anxiety behavior^37^, suggesting a possible common substrate critical for the control of dopaminergic neurons hyperactivity. Restoring a physiological balance in calcium channel gene expression and activity may reduce the calcium overload in the DA terminals, an important consideration that should be taken into account for the treatment of symptomatic PD patients.

Our examination of PPR confirmed that the major presynaptic change induced by the A53T mutation was confined to the DLS, where the absence of facilitation of PreCaTs indicates a substantial saturation of cytosolic [Ca^2+^] in response to initial action potential firing and terminal activation. VS terminals showed signs of reduced facilitation of calcium in response to paired pulse activation of terminals in the A53T mutant mice, perhaps indicative of pathology caused by the mutation without being symptomatic in terms of synaptic efficiency. For the interpretation of these results, it must be considered that the A53T model used in this study induces a progressive neurodegeneration of SNc, but not VTA, mDANs together with an early and severe axonal deramification that remains stable throughout the aging process^5^. The calcium responses in the surviving axons might be a consequence of the mutant α-synuclein accumulation, a sort of insult that the degenerating smaller fibers could not tolerate. The resilience of these surviving fibers directs our attention to understanding the origin of such endurance. We found that aldehyde dehydrogenase 1a1 (ALDH1A1), may be a good candidate for a molecular marker of PD-resistance^38,39^. In human PD sufferers, ALDH1A1-positive mDA neurons disappear along the PD progression, but in our A53T mutant mice the same neurons are resistant to α-synuclein insults^40^. The correlation between DA release and PreCaTs (Fig. 4F) from DLS of control mice suggests the existence of two clusters of recordings, one of which showed highly correlated DA release/PreCaT ratios, as observed in A53T mutants. Interestingly, the reduction of small puncta in A53T mice is associated with the loss of recording fields in which the DA release is independent from the PreCaTs amplitudes. This might be related to the loss of ALHD1a1-negative DA fibers in the DLS. In fact, we observed that in the matrix of wild-type mice DA release was augmented compare to the ALHD1a1-enriched striosomes^38^. It remains to be determined if ADLH1A1-expressing DA projections show the same resilience, if the unbalance between N- and P/Q-type channel activities presented in this paper are related to ALDH1A1 expression, and ALDH1A1 can be targeted together with the available therapeutic approaches for improving life expectancy of PD patients.

## Methods

### Transgenic Mice

Transgenic mice were generated as previously reported in detail^7,11^. Heterozygous tetO-GCaMP3 [Tg(tetO/Prnp-GCaMP3)600Cai, JaxLab stock no, 027783, C57BL/6J strain background], tetO-A53T mice [Tg(tetO-SNCA*A53T)E2Cai, JaxLab stock no. 012442, C57BL/6J strain background] and Pitx3-tTA [B6.129(FVB)-Pitx3tm1.1Cai/J, JaxLab stock no. 021962, 95% C57BL/6J strain background] were crossed together, housed in a 12-h light/dark cycle and fed regular diet ad libitum. 3–5 month old control mice (Pitx3-tTA:tetO-GCaMP3 or Pitx3/GC3) and A53T mutants (Pitx3-tTA:tetO-GCaMP3:tetO-A53T mice) were used in this work. All mouse work follows the guidelines of the Institutional Animal Care and Use Committees (IACUCs) of National Institutes of Health, and was approved by the ACUCs of National Institute of Alcoholism and Alcohol Abuse and National Institute on Aging.

### Genotyping

Genomic DNA was prepared from tail biopsy using DirectPCR Lysis Reagent (Viagen Biotech) and subjected to PCR amplification using specific sets of PCR primers for each genotype, including Pitx3-tTA transgenic mice (Pitx3-F, GAC TGG CTT GCC CTC GTC CCA; Pitx3-R, GTG CAC CGA GGC CCC AGA TCA) and tetO-GCaMP3 mice (GCaMP-F, TAC TGC TCC ATT TTG CGT GA; GCaMP-R, TTG CTG TCC ACC AGT CAT GC), and tetO-A53T (SNCA -F, TAC TGC TCC ATT TTG CGT GA; SNCA-R, TCC AGA ATT CCT TCC TGT GG).

### Slice Preparation and Dual Voltammerty/Photometry Recording

Following isoflurane anesthesia, brains from 10–14 week mice were removed and 250-μm-thick coronal sections through the striatum were prepared in carbogen-bubbled, cold high-sucrose solution (in mM: Sucrose 194, NaCl 30, KCl 4.5, MgCl_2_ 1, NaHCO_3_ 26, NaH_2_PO_4_ 1.2, Glucose 10) [18]. Slices were then transferred to a chamber filled with oxygenated artificial cerebrospinal fluid (aCSF; (in mM: 126 NaCl, 2.5 KCl, 1.2 NaH_2_PO_4_, 2.4 CaCl_2_, 1.2 MgCl_2_, 25 NaHCO_3_, 11 glucose, 20 HEPES, 0.4 L-ascorbic acid) at 32 °C and allowed to recover for 1 h. Slices were then pre-incubated at room temperature until used for recording. Dual voltammetry and photometry recording procedures were followed as previous reported^11^. Briefly, hemisections were transferred to a recording chamber and constantly superfused with aCSF at 29–31 °C at a rate of 1.5 ml/min using a peristaltic pump. A bipolar concentric electrode was positioned in close proximity to the recording field (in the overlying white matter for DLS, in the border between core and shell of Nucleus Accumbens for VS). Regions of interest (ROI: 180 µm × 180 µm in a 250-µm thick slice) where defined in DLS and VS (Nucleus Accumbens, Shell) and a carbon fiber was inserted at the ROI center. Electrical stimulation was applied to activate populations of axon fibers within the ROIs considered, by rectangular, electrical pulse stimulation (120 µA, 1.2msec, monophasic, unless otherwise noted) using an isolated constant current stimulator (DS3/GG2A system Digitimer Ltd, Hertfordshire, UK), and delivered every 5 min. For FSCV/photometry simultaneous recordings, two systems were synchronized to the same electrically evoked event. Fluorescence in ROIs was excited using light emitted by a mercury burner (Zeiss FluoArc Variable Intensity Lamp Control for HBO 100, Carl Zeiss Microscopy GmbH, Jena, Germany) and the exposure was controlled by a shutter (model V25; Uniblitz, Vincent Associates, Rochester, NY). The light emitted from the ROI was filtered at 535 nm and sent to a photomultiplier tube (PMT; Model D-104, Photon Technology International, Edison, NJ, USA). The PMT voltage output (time constant: 5 ms; gain: 400 × 10–1 µA/V) was fed into a computer interface (Digidata 1322A, Axon Instruments, Molecular Devices LLC, Sunnyvale, CA). Data were sampled at 100 Hz and stored on a PC hard drive using PCLAMP 9.2 software (Axon Instruments, Molecular Devices LLC, Sunnyvale, CA). For FSCV, the carbon-fiber electrode was held at −0.4 V, and the potential was increased to 1.2 V and back at 400 V/s every 100 ms using a triangle waveform. Data collection and analysis were performed using the Demon Voltammetry and Analysis software suite^41^.

Analysis was performed offline using cursor-based measurements in Clampfit (pCLAMP 9.2 software, Molecular Devices LLC, Sunnyvale, CA), while FSCV data were analyzed using DEMON software (Winston-Salem, NC). As previously described, both sets of data were mathematically corrected for drift caused by photobleaching and UV light – carbon fiber scan interference^11^. Calcium transients were measured as the ratio of the peak amplitude (ΔF) to the averaged baseline fluorescence value (F) measured before the stimulus. For DA release, ten cyclic voltammograms of charging currents were recorded as background before stimulation, and the average of these responses was subtracted from data collected during and after stimulation. I/O curves were constructed by plotting stimulus current versus response amplitude over a range of stimulus intensities or number of pulses. For paired-pulse ratio, single and paired stimulation of defined inter-stimulus intervals were alternated. Then the average of single pulse transients before and after the paired one were calculated and subtracted from the paired pulse stimulation transient to define the second peak amplitude. Two-way ANOVA were used for statistical comparisons between groups. For pharmacological experiments, fluorescent transients were expressed as a percentage of the average of the first 10 min of pre-drug control baseline, and compared to non-treated conditions, to account for the slight time-dependent decrease in the amplitude of the transient in the absence of treatment. Two-way ANOVA and MANOVA were used for statistical comparisons between different conditions. For better representation of the data, Bonferroni’s Post Hoc tests shown in Figures were applied to data collected during drug treatments.

### Drugs

ω-conotoxin GVIA (CTX), ω-agatoxin IVA (ATX), nifedipine, quinpirole and sulpiride, were obtained from Sigma-Aldrich. (1S, 2S)-2-[2-[[3-(1H-Benzimidazol-2-yl) propyl] methylamino]ethyl]-6-fluoro- 1, 2, 3, 4-tetrahydro-1-(1-methylethyl)-2-naphthalenyl cyclopropanecarboxylate dihydrochloride (NNC 50-0396), Cyclopiazonic acid (CPA) and dihydro-β-erythroidine (DHβE) were obtained from Tocris Cookson. Drugs were dissolved as stock solutions in water or DMSO and aliquoted and frozen at −20 °C before use. Each of the drugs was diluted in aCSF immediately before each experiment. When used, the final concentration of DMSO external solution was always <0.05%.

### Immunohistochemistry

Tissues from *ex-vivo* coronal slice preparation of Pitx3/GC and Pitx3/GC/A53T mice were also used for histological analysis. Slices were fixed overnight with 4% formaldehyde in PBS, beginning at room temperature for at least 30 min and then sections were transferred to +4 °C. After washing overnight in PBS plus 0.2% triton X-100 (PBST), the slices were rinsed in deionized water (dH_2_O), then incubated twice for 10 min in freshly prepared sodium borohydride (5 mg/m: in dH_2_O). After rinsing again in dH_2_O, sections were incubated in 0.2% Sudan Black in 70% ethanol, for 15 min room temperature (RT)^42^. Slices were then destained twice for 30 min in PBS RT, and then incubated for at least 4 h in 5% BSA in PBST. Antibodies specific to green fluorescent protein (GFP; 1:2000; Sigma-Aldrich), Tyrosine Hydroxylase (TH; 1:2000, Dynal Biotech) and human alpha-synuclein mutation A53T (syn211, 1:500; Santa Cruz Biotechnology) were used for incubation over the weekend at +4 °C. After three rinses in PBS, Alexa Fluor 430- (Cat #A-11063, 064), 488- (Cat #-11034) or 568- (Cat# A-11004, 011) conjugated secondary antibodies (1:500; Invitrogen) was used for incubation for 24 h. Fluorescence images were captured using a stereoscope (SteREO discovery, Zeiss, Thornwood, NJ) and a laser scanning confocal microscope (LSM 510; Zeiss, Thornwood, NJ). The stereoscopic images were collected using light emitted by a mercury burner filtered at 535 nm set at 12x magnification, using the same gain, focus from the surface and offset settings for each slice using AxioVision software (Rel. 4.8., Zeiss, Thornwood, NJ). The confocal images were collected using LSM imager or Leica LCS software (Zeiss, Thornwood, NH), as single optic layers with a 100×/1.4 NA oil immersion objective. An excitation filter of 480/35 and emission filter of 535/30 were used for green fluorescence, while an excitation filter of 540/25 and emission filter of 605/55 were used for red fluorescence. Post-collection processing was applied uniformly to all paired images using ImageJ (http://imagej.nih.gov).

### Image analysis

The images were analyzed as single optic layer. For the quantitative assessment of GFP marker protein distribution in striatum samples, images were taken using identical settings and exported to ImageJ (NIH) for imaging analyses. Images were converted to b/w color scale (fluorescence intensity from 130 to 255) using ImageJ. Using the 100x magnification, four 90 × 90 µm areas of interest were first selected in three properly coronal sections (from Bregma 1.18 mm to 0.86 mm). From the same samples, five 25 × 25 µm areas of interest were selected pseudo randomly, without included cortico-striatal fibers regions, and then subjected to measurement by mean particles analysis. Student’s t test and two-way ANOVA was used to compare different groups.

### Laser Capture Microdissection (LCM) and quantitative reverse transcription polymerase reaction (qRT-PCR)

Mice were euthanized by overdose with isoflurane and the brains were quickly dissected out and frozen at −80 °C. The frozen mouse brains were then sectioned with a cryostat at −20 °C and loaded onto the PAN membrane frame slide (Applied Biosystems, Foster City, CA). The mDANs were dissected by the LCM with the ArturusXT micro-dissection system (Applied Biosystems) based on the presence of green fluorescent proteins (GFP) as described previously^7^. Briefly, the total RNA was extracted from around 800 isolated mDANs per mouse brain with the PicoPure Isolation kit (Applied Biosystems) and the cDNAs were synthesized with the First Strand kit (QIAGEN, Valencia, CA) from equal amounts of total RNAs in the comparison groups. The SYBR Green real-time PCR detection method was used to quantitate the calcium channel expression. All the primers used in the qPCR were from QIAGEN and tested by the manufacturer. The expression levels were calculated as fold changes normalized with the β-actin. Data were statistically compared with unpaired t test, two tailed.

## Supplementary information

Supplementary Figures
