# Supplementary material for: Unbalanced calcium channel activity underlies selective vulnerability of nigrostriatal dopaminergic terminals in Parkinsonian mice
Source: Sci Rep. 2019 Mar 19;9:4857. doi: 10.1038/s41598-019-41091-7 (PMC6425036; doi:10.1038/s41598-019-41091-7)
Supplement: Supplementary file 1 — Supplementary Figures [file 41598_2019_41091_MOESM1_ESM.docx]

**Unbalanced calcium channel activity underlies selective vulnerability of nigrostriatal dopaminergic terminals in Parkinsonian mice.**

Carmelo Sgobio^1, 2^, Lixin Sun^1^, Jinhui Ding^3^, Jochen Herms^2^, David M. Lovinger^4, *^, and Huaibin Cai^1, *^

**Supplementary Figures and Figure Legends.**

**Fig. S1**

**
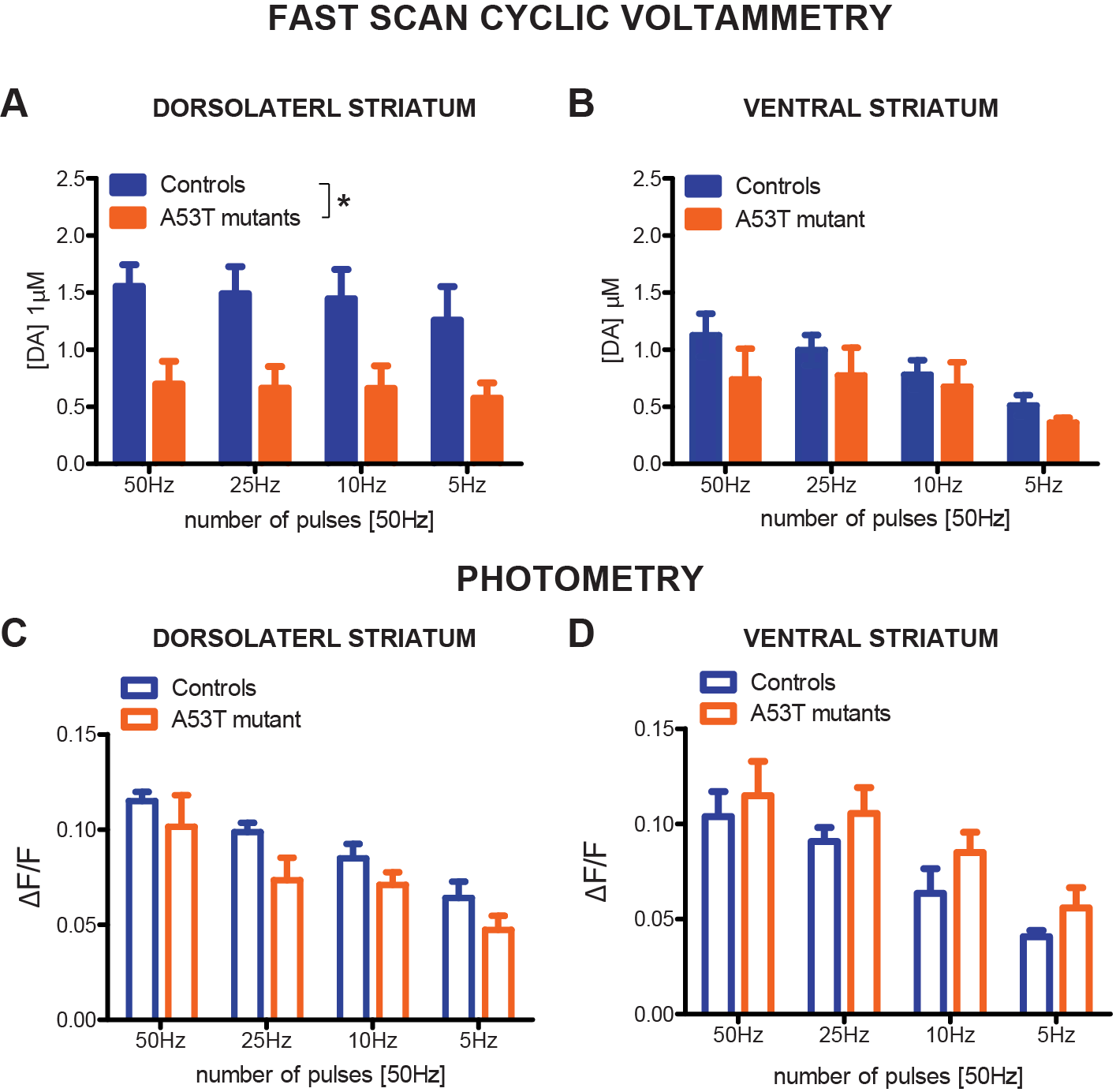
**

**Fig. S1 Reduction in DLS DA release, but no change in PreCaTs evoked by 6-pulse burst stimulation at different frequencies in A53T mutants versus controls.** (A) DA release in both genotypes was comparable at all stimulus frequencies in DLS but was lower at all frequencies in A53T mutant mice (Two-way ANOVA Genotype MAIN FACTOR F(1,7) = 6.254, p<0.05; Frequency MAIN FACTOR F(3,21) = 5.166, p< 0.01 ). (B) In VS, DA release increased with increasing frequencies in both genotypes, with comparable release in both genotypes at each frequency (Two-way ANOVA Frequency MAIN FACTOR F(3,21) = 13.39, p<0.001). On the other hand, photometry recordings showed no difference in concurrently measured PreCaTs due to genotype in DLS (C) (Two-way ANOVA Frequency MAIN FACTOR F(3,21) = 30.54, p<0.001or VS (D) ( Two-way ANOVA Genotype MAIN FACTOR F(3,24) = 47.44, p<0.001).

**Fig. S2**

**
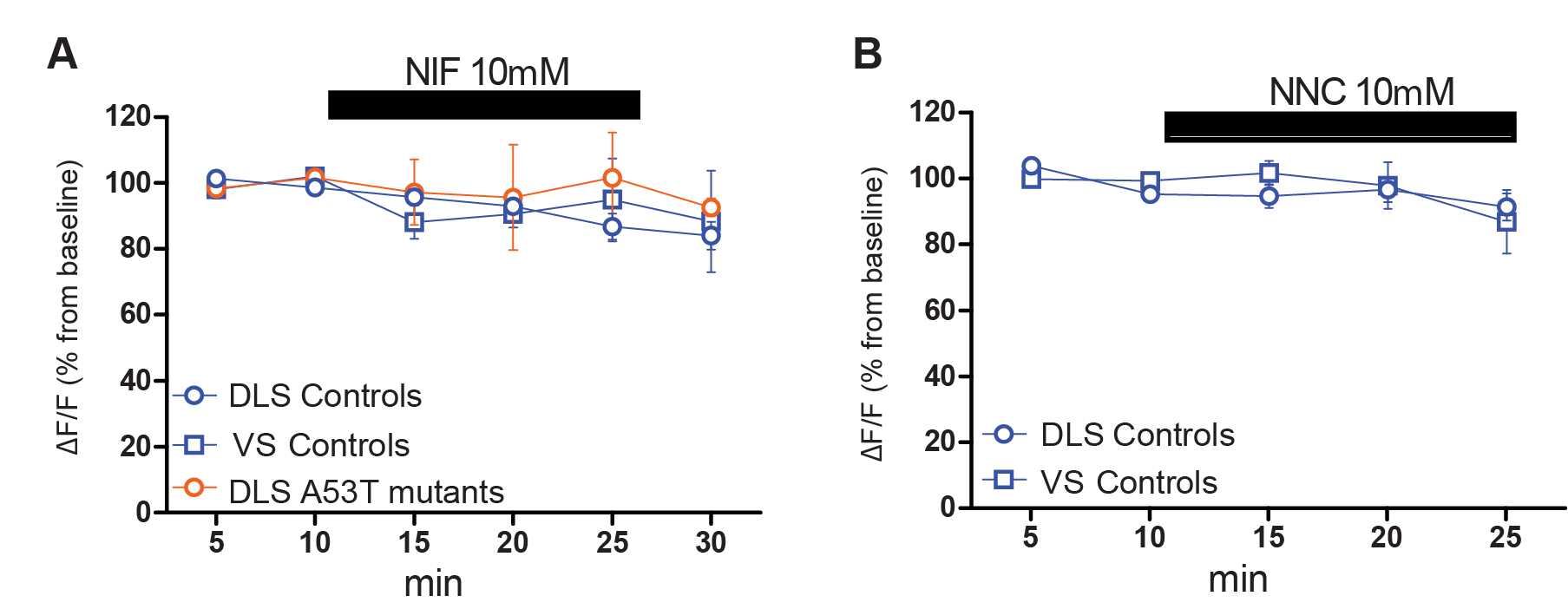
**

**Fig. S2 L- and T-type calcium channels showed no significant role in DA terminal PreCaTs under single pulse stimulation protocol.** (A) Nifedipine (NIF), an L-type channel blocker, did not affect PreCaTs in either striatal region of control mice or A53T mice. (B) Application of the highly selective T-type calcium channel blocker NNC 55-0396 dihydrochloride (NNC) had no effect on PreCaTs in either control or A53T slices from both striatal regions (Repeated measures ANOVA not significant (DLS: F(4) = 2.478, p>0.05; VS: F(4) = 1.476. p>0.05).

Fig. S3


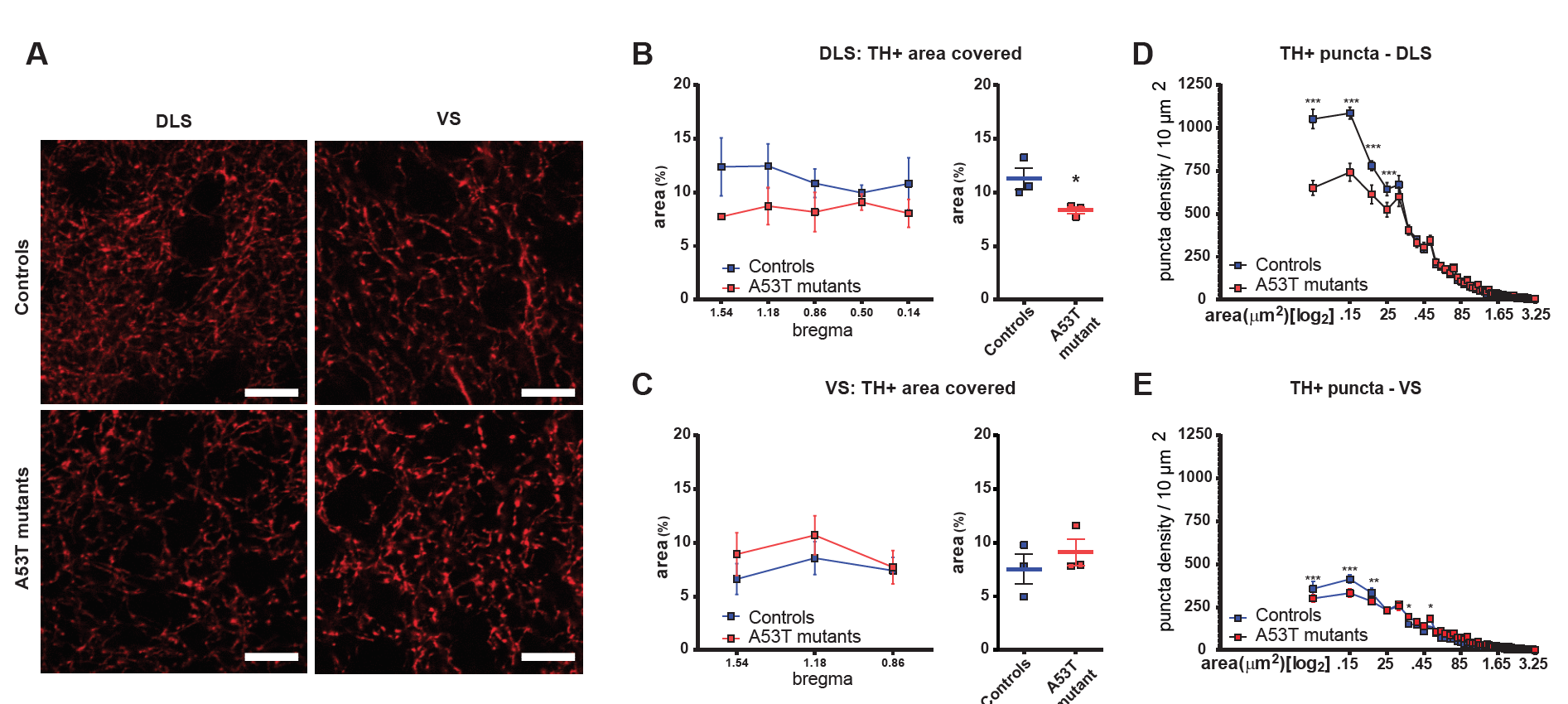


**Fig. S3 TH-positive fiber striatal innervation is reduced in DLS but not in VS of A53T mutants.**

(A) Representative confocal images of TH-positive dopaminergic fibers in DLS. Scale bar: 5 µm. DLS (B) and VS (C) average area covered by TH-positive fibers in consecutive coronal sections of striata (left) and in total (right). Only in DLS, the area covered by TH-positive fiber was significantly lower than controls (Student’s t test: t_(54)_=2.826, p<0.05). (E) A significant reduction of observed puncta frequencies, smaller than 0.25 µm^2^, was detected in DLS of A53T mutants (Two-way ANOVA Genotype x Size Interaction Factor F_(63,256)_ = 10.25, p<0.001; Bonferroni’s *post hoc* test: ***p<0.001). In VS of A53T mutants, less small size puncta (**<**.20 µm^2^) but more middle size (between .35 and .5 µm^2^) were detected (Interaction Factor F_(63,256)_ = 2.778, p<0.001; Bonferroni’s *post hoc* test: *p<0.05, **p<0.01,***p<0.001).
